# Bac*Dive* in 2022: the knowledge base for standardized bacterial and archaeal data

**DOI:** 10.1093/nar/gkab961

**Published:** 2021-10-29

**Authors:** Lorenz Christian Reimer, Joaquim Sardà Carbasse, Julia Koblitz, Christian Ebeling, Adam Podstawka, Jörg Overmann

**Affiliations:** Leibniz Institute DSMZ-German Collection of Microorganisms and Cell Cultures, Braunschweig, Germany; Leibniz Institute DSMZ-German Collection of Microorganisms and Cell Cultures, Braunschweig, Germany; Leibniz Institute DSMZ-German Collection of Microorganisms and Cell Cultures, Braunschweig, Germany; Leibniz Institute DSMZ-German Collection of Microorganisms and Cell Cultures, Braunschweig, Germany; Leibniz Institute DSMZ-German Collection of Microorganisms and Cell Cultures, Braunschweig, Germany; Leibniz Institute DSMZ-German Collection of Microorganisms and Cell Cultures, Braunschweig, Germany

## Abstract

The bacterial metadatabase Bac*Dive* (https://bacdive.dsmz.de) has developed into a leading database for standardized prokaryotic data on strain level. With its current release (07/2021) the database offers information for 82 892 bacterial and archaeal strains covering taxonomy, morphology, cultivation, metabolism, origin, and sequence information within 1048 data fields. By integrating high-quality data from additional culture collections as well as detailed information from species descriptions, the amount of data provided has increased by 30% over the past three years. A newly developed query builder tool in the *advanced search* now allows complex database queries. Thereby bacterial strains can be systematically searched based on combinations of their attributes, e.g. growth and metabolic features for biotechnological applications or to identify gaps in the present knowledge about bacteria. A new interactive dashboard provides a statistic overview over the most important data fields. Additional new features are improved genomic sequence data, integrated NCBI TaxIDs and links to Bac*Media*, the new sister database on cultivation media. To improve the findability and interpretation of data through search engines, data in Bac*Dive* are annotated with bioschemas.org terms.

## INTRODUCTION

Microorganisms drive the global biogeochemical cycles, cause a large variety of diseases and constitute an underexplored resource for biotechnological applications. Our primary knowledge on bacteria and archaea still relies on mostly cultivating and describing isolated strains, from which there are currently 17 470 species with validly published names under the ICNP (International Code of Nomenclature of Prokaryotes, https://lpsn.dsmz.de/text/numbers, accessed July 2021) and could be studied in detail. Yet, this number represents only a very small portion of the existing prokaryotic diversity, estimated to range from 0.1% to 0.001% (1), while many of the remaining prokaryotes that can be detected by culture-independent metagenomic sequencing will have to wait for long before becoming available for characterization in laboratory cultures.

The rapid increase in heterogeneous and highly distributed data for the described prokaryotic species and the need for phenotypic predictions from genome sequences of not-yet-cultivated ones require a more comprehensive data integration and an improved analysis of phenotype-genotype relationships, particularly through the application of artificial intelligence (AI) methods that have gained increasing importance recently. The precision of predictions made by AI is highly dependent on the amount and the quality of the data to train its algorithms. In cases of good data availability, AI has proven to be superior to the human intellect that is not able to digest and process huge amounts of data (2,3) and first attempts to apply AI-methods to predict traits for microorganisms (4–6) have demonstrated how high quality, standardized and digitally available data for described microorganisms can help us to access the vast majority of so far unexplored diversity.

Until today, most of the knowledge about microorganisms is hidden in publications, private lab notebooks as well as internal databases of culture collections. Bac*Dive* represents the worldwide largest database for standardized prokaryotic information. Its mission is to mobilize and integrate research data on strain level from diverse sources and make it freely accessible. So far, Bac*Dive* is the only source that enables a profile search - to find and analyze bacterial and archaeal strains based on their specific attributes. Here, we report about new content and major recent improvements in functionalities of Bac*Dive*.

## CONTENT

The backbone of Bac*Dive* is the high-quality data retrieved from internal files from culture collections, e.g. the German culture collection (Leibniz Institute DSMZ) and the collection at the institute Pasteur (CIP, France). In recent years, several other collections have joined, including the Swedish culture collection at the University of Gothenburg (CCUG) and the Centre for Agriculture and Bioscience International (CABI), which has resulted in an increase of data for 19 223 additional strains. Compared to the number of strains covered by Bac*Dive* in 2018 (63 669) (7) this represents a growth of 30%. The number of type strains has increased by 1493 in the same time. With overall 14 208 type strains, Bac*Dive* currently covers 81% of the total of 17 470 species with validly described names (https://lpsn.dsmz.de/text/numbers, accessed July 2021).

Primary literature, especially species descriptions are still the most valuable but also most challenging source for data on prokaryotic species. Missing standardization, e.g. for metabolic names, but also surprising creativity in the reporting of plain experimental results, as well as inconsistencies in the data impede a reliable automated extraction. Our ongoing approach to annotate data manually from species descriptions resulted in 6898 type strains with expanded datasets that offer standardized data for up to 152 data fields. This represents a growth of 26% compared to the status reported in 2018. Already 49% of all type strains in Bac*Dive* are enriched by comprehensive data from literature. To keep up with the increasing speed of new species descriptions published, a semi-automated workflow is in development which will improve not only annotation speed drastically, but makes standardized data of newly described species rapidly available in Bac*Dive* shortly after publication.

In 2019, the database *List of Prokaryotic names with Standing in Nomenclature* (LPSN) moved to the Leibniz Institute DSMZ and was merged with the DSMZ service *Prokaryotic Nomenclature Up-to-Date* (PNU) (8). Technically, the database was newly constructed and became a sister-database for Bac*Dive*. Since then, the data on nomenclature provided by Bac*Dive* is updated on a regular basis using correct names from LPSN. For better usability, taxonomic information in Bac*Dive* links to LPSN and type strain information in LPSN links to Bac*Dive* (where available).

Cultivation media are of prime interest for microbial researchers, as they are the basis for cultivating newly isolated species. Bac*Dive* currently offers 66 570 data sets on cultivation media for 36 169 strains. 30 108 (45%) of the media recipes originate from the DSMZ collection. With 1893 different cultivation media recipes, this represents the worldwide largest collection for microbial cultivation. In the past, media recipes from the DSMZ were provided as PDF files, which made them difficult to access and analyze. Therefore, the new database Bac*Media* (https://bacmedia.dsmz.de/) was created as another sister-database of Bac*Dive*, offering DSMZ media recipes in a standardized format and specialized functions around cultivation media. Cultivation media descriptions in Bac*Dive* are updated through Bac*Media* (and linked), while media recipes in Bac*Media* are linked to those strains in Bac*Dive* that are able to grow on these. Additionally, metadata from Bac*Dive* are integrated in Bac*Media* in a media-centralized manner and link to the Bac*Dive* advanced search.

**Figure 1.**
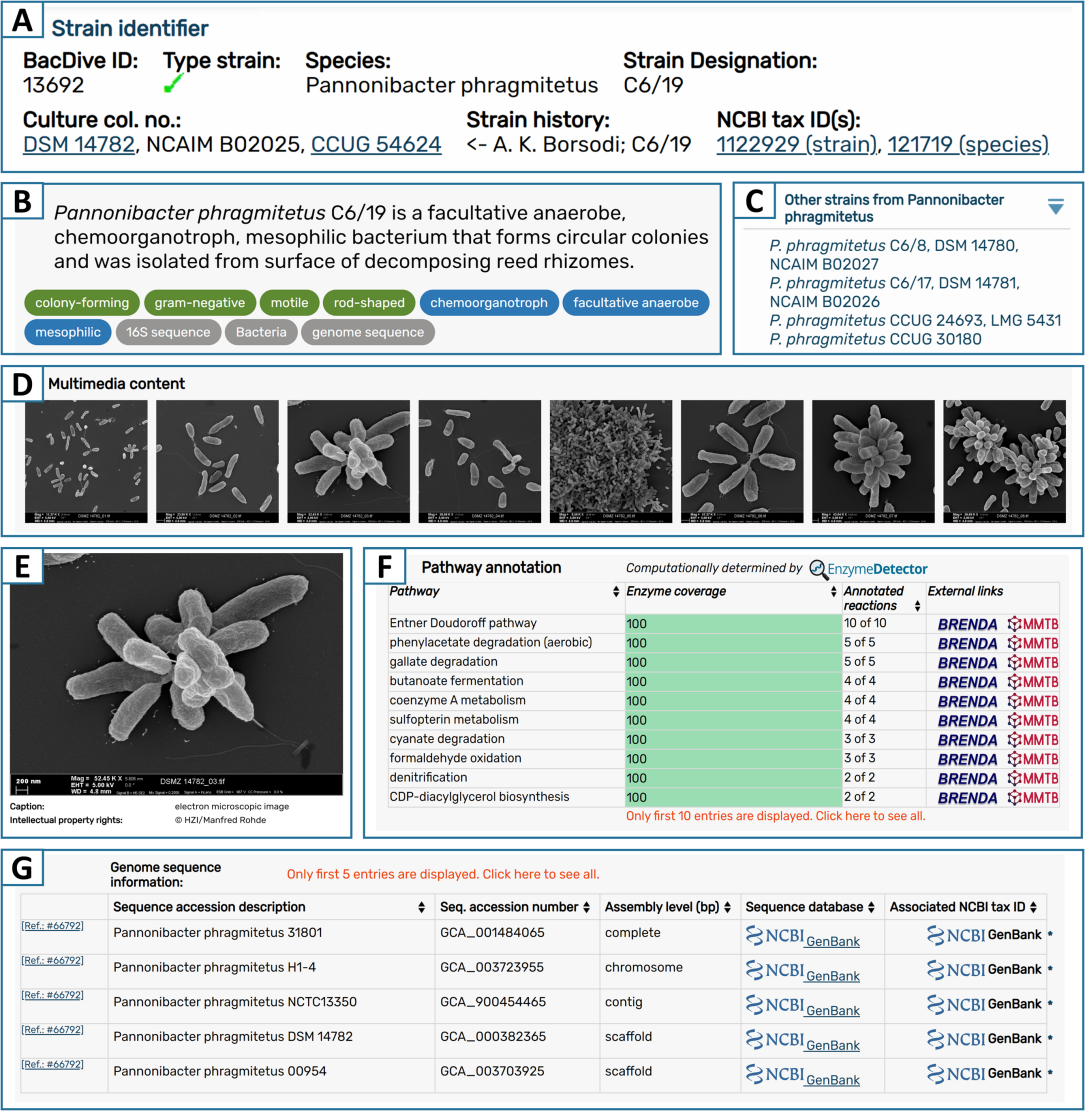
Updated features in the strain detail view. (**A**) Infobox comprising main strain identifier, including BacDive ID, culture collection numbers and NCBI taxonomy ID. (**B**) Short summary of the most important information on the strain. (**C**) Species navigation bar, to switch between strains that belong to the same species. (**D**, **E**) Gallery and detail view for multimedia content, e.g. electron microscopy images. (**F**) Metabolic pathway information integrated from EnzymeDetector, displayed in a list sorted descending by the coverage of detected enzymes. (**G**) Table with genomic sequence data retrieved from NCBI GenBank, PATRIC and JGI.

As Bac*Dive* is designed as a metadatabase, sequence data itself are explicitly not provided. Still information about available sequences connected to a strain, especially 16S rRNA and genome sequence data are of utmost importance to serve the key mission of Bac*Dive* to enable phenotype-genotype comparisons. Therefore, the *molecular biology* section was renamed to *sequence information* and restructured to provide 16S rRNA and genomic sequence information in distinct data fields. The data for 16S rRNA and genomic information were updated recently. For this purpose, we retrieved genome sequence metadata from NCBI GenBank (9), PATRIC (10) and JGI (11), and matched the data via culture collection numbers and NCBI taxonomy IDs (Figure 1G). This led to an increase in this section from 30 475 entries to 295 734 since 2018, which is largely due to an increase in genomic sequence information. For 9966 strains, an average of 30 genomic assembly datasets are offered per strain, giving the user a good overview about the available genomic assemblies provided by the three databases. Furthermore, the matching of NCBI taxonomy IDs (12) is included as a new data field on both, species and strain-level (Figure 1A).

Although the Bac*Dive* database focuses on providing experimental data and observations, gene annotation data provide another important source of information. In order to give users a fast overview about the metabolic capabilities, data from the database EnzymeDetector (13) were integrated into the datasets of 7048 strains. EnzymeDetector is a tool provided by the database BRENDA that integrates enzyme annotation data from various resources. In Bac*Dive*, the EnzymeDetector data were condensed into pathways, which are displayed in a list of detected enzymes sorted in descending order by the coverage of the pathway (Figure 1F). Every pathway links to the BRENDA pathway maps as well as the Metano Modeling Toolbox providing detailed information on the enzymatic reactions involved.

Profiles of microorganism are usually completely data driven and visual representations are only rarely available. Still, images are essential to illustrate cell morphology and arrangement. The already provided 1213 microscopic images on myxobacteria from the Reichenbach collection (14) were now augmented by 2493 light microscopic images as well as macroscopic images from agar plates originating from the DSMZ. With the latest Bac*Dive* update, 1499 electron microscopy images captured by Manfred Rohde at the Helmholtz Centre for Infection Research (HZI) were integrated, which provide a detailed insight in the morphology and arrangements of bacterial cells (Figure 1D, E).

## USER INTERFACE

The Graphical User Interface (GUI) of Bac*Dive* is built with an individually developed PHP front end, combined with 108 tables stored in a MariaDB SQL database. This setup ensures the flexibility to keep up with the ever-evolving database and allows to integrate new data types and to adapt the GUI according to user feedback and needs. The biodiversity provided in Bac*Dive* is also reflected in the diversity of data and thereby in the growing number of data fields, which has increased by 75% to an overall of 1,048 since the last report. Although the potential diversity of data is high, typically only a fraction of the data fields is occupied for individual strains. Therefore, the Bac*Dive* GUI displays only filled data fields, which is why especially new users that only have looked up single strains, do not perceive the potential of data that is available for other strains (see dashboard).

### Home page

Since the beginning, the Bac*Dive* home page (https://bacdive.dsmz.de/) provides a *Simple search* to find the strain of interest easily either by its culture collection number or the name of the species. Over time, the home page was amended with more information and links to a growing number of subpages. Therefore, the home page was completely redesigned and the *Simple search* improved. The new *Simple search* offers extended search options like Bac*Dive*-ID, NCBI taxonomy ID and 16S sequence accession number. Once the user clicks on the search field, examples for all available search methods are shown. Moreover, the new home page offers several other novel features. A gallery shows example data from the newly developed statistics-dashboard. Condensed information on an example strain is shown, to give new users a starting point to learn more about Bac*Dive*. The latest news is displayed to inform users about recent developments.

### Dashboard

**Figure 2.**
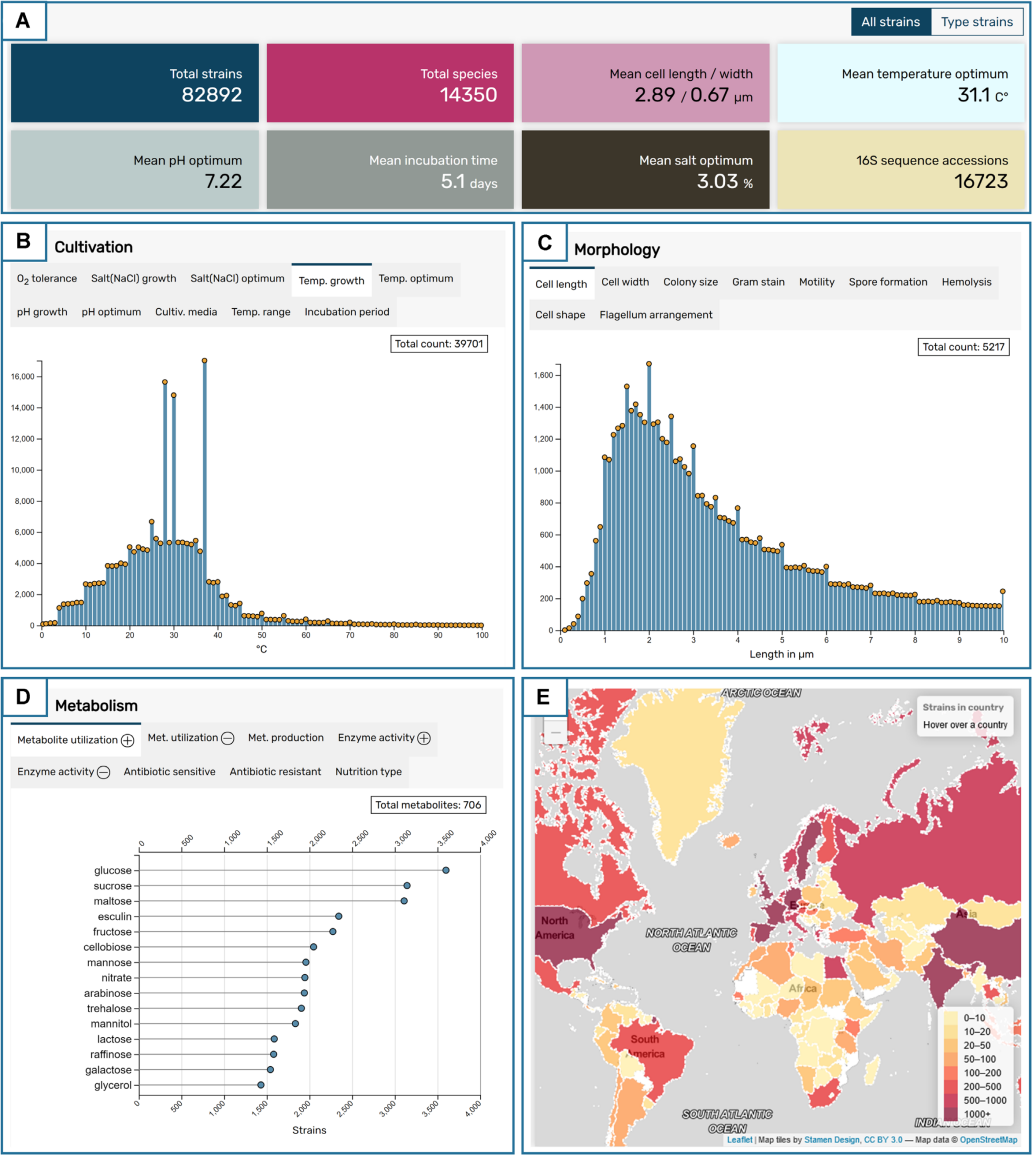
Selected statistics from the dashboard (https://bacdive.dsmz.de/dashboard) providing an overview over Bac*Dive* data within 49 graphs. (**A**) Eight key values covering total strains and species, cell size, optimum cultivation temperature, pH, incubation time, optimum salt condition, and count of 16S sequence accession numbers. (**B**) Cultivation data showing distribution of growth temperatures for 39 701 strains. (**C**) Morphology data showing distribution of cell length for 5217 strains. (**D**) Metabolism data showing a ranking of 706 utilized metabolites ordered descending by strain count. (**E**) Location data showing the number of strains that were isolated in each country.

With 1048 data fields, Bac*Dive* offers a high diversity of data that can be hardly perceived by new users. To give users an overview of the Bac*Dive* data offer, a dashboard was developed that provides statistical information for various aspects of data (Figure 2, https://bacdive.dsmz.de/dashboard/). At the top of the page, eight values are displayed giving information about the total numbers of strains, the number of species, mean values for cell size, optimal temperature, optimal pH, incubation time, optimal salt concentration and the count of 16S sequence accession numbers. In the top right, a switch allows the user to restrict the displayed statistics to data from type strains, which reflects species information more objectively. Below, 49 graphs are shown that are grouped in eight tiles, covering the fields taxonomy, morphology, cultivation, metabolism, isolation source, molecular biology, pathogenicity and physiological tests. Data are displayed interactively as bar charts, pie charts or on a world map (Figure 2B–E). By clicking on single data points, a corresponding search is started, either in the advanced search, the isolation source search, or the TAXplorer, depending on the data field. In this way, the dashboard offers the ideal starting point for exploring the data in Bac*Dive*.

### Advanced search

With 142 search fields in eleven categories, the *Advanced search* (https://bacdive.dsmz.de/advsearch) represents the most powerful tool to query the Bac*Dive* database. Previously, the high number of search fields represented a challenge for new users to get an overview of the options and to find the best way to retrieve the information of interest. Furthermore, the previous version of the *Advanced search* was only able to query each search field once, which drastically limited the dimensions of search options. For this reason, a completely new *Advanced search* was developed, offering a query builder as well as comfort functions to get a better overview of the data fields. At the top left of the page, the new query builder allows to query up to three groups, of which each group can accommodate up to five single queries. Within the groups, search fields are connected by an ‘AND’ operator, while between the groups the operator can be switched between ‘AND’ and ‘OR’. When choosing a field, a well-sorted overview of the available search fields is shown on the right, allowing users to select a field easily. When the user starts typing in a text search field, an auto-complete function suggests possible entries for the queried data field. By inserting a wildcard (‘*’), the search returns all available entries. Every search field can be moved within the query builder by *drag & drop*, even between groups. Below the query builder, example queries are given that demonstrate the potential of the *Advanced search* by answering possible questions of interest. Examples can be selected and used as a starting point to build own queries. The search result is then provided on the right side, which allows to step-wise refine a query until the desired result is returned. The performed search can be saved by bookmarking or shared via the ‘copy query link’ function. To retrieve the data, two options are available. First, the resulting strains can be added to a download selection, which allows to customize the exported data, but is limited in the number of strains. Second, the queried data can be directly downloaded as a CSV file, which contains all the data displayed in the search result.

### Isolation source search

Usually isolation sources are described in highly diverse ways, ranging from single words like ‘soil’ to long sentences describing every detail the researcher has observed during sampling. To get a systematic access to the origin of microbial strains, the *Isolation source search* was developed (https://bacdive.dsmz.de/isolation-sources). The central parts of this tool are *Isolation source tags*, which are keywords that were manually assigned to each isolation source. The keywords are organized in a hierarchical ontology that comprises 387 terms on three levels. By selecting keywords from the different levels, the broadness of the information can be adjusted, e.g. in the order #Environmental > #Aquatic > #Marine. By combining different keywords, even complex isolation sources can be described or selected. Besides filtering isolation sources based on their *Isolation source tags*, the search tool provides several other functions, e.g. filtering by country, host or taxonomic data. Resulting strains can be displayed on a world map and the assigned keywords can be analyzed in a Krona-plot. Like the results of the advanced search, data can be retrieved either via the download selection or directly downloaded as a CSV file.

### Strain detail view

The strain detail view provides all information about a strain (Figure 1). While the data are arranged in nine thematic sections, strain identifiers like species, strain designation, culture collection numbers, and NCBI taxonomy IDs are displayed in an info box at the top of the page (Figure 1A). To access the most important information about a strain at the first glance, a human readable short summary, together with a couple of keywords is presented above the sections. To easily navigate within one species, short links to other strains from the same species are provided in the navigation bar at the top right. Within the *sequence information* section, a new field was integrated that offers access to the output of the tool protologger (http://www.protologger.de) (15). Protologger is a genome description tool that streamlines the process of gathering genomic data on taxonomic, functional and ecological information. Therefore, this information perfectly amends the physiological data provided by Bac*Dive* (an example is given here: https://bacdive.dsmz.de/strain/159029).

## DATA ACCESS

The GUI is only one way to access the data in Bac*Dive*. Machine-readable and interpretable data are becoming more and more important. Representing information in a machine-interpretable format, such as the resource description framework (RDF), will enable algorithms to understand and answer questions based on the knowledge stored in databases. So far, search engines use already standardized vocabularies (https://schema.org/) that enable basic queries. The Bioschemas-initiative (https://bioschemas.org/) aims to extend this vocabulary with scientific information. Bac*Dive* has now integrated first Bioschemas representations to further improve the findability of information in Bac*Dive* data through search engines.

For large-scale analyses, data can be retrieved using the RESTful web service of Bac*Dive* (https://api.bacdive.dsmz.de/). First established in 2015, this service was now completely reconstructed, offering new functionality and improved expandability for the future. For the comfort of the users, a detailed documentation is offered as well as example clients in R and Python (https://api.bacdive.dsmz.de/client_examples).

The Bac*Dive* web service is free of charge, but needs a registration. To facilitate access for the users, who often have to handle a large number of authentication credentials, a new authorization system was established based on the keycloak software (https://www.keycloak.org/). This system serves as a central authorization server, enabling access to all DSMZ web services, which includes Bac*Dive* as well as the new LPSN web service (https://api.lpsn.dsmz.de/) and all future services. More importantly, this system enables single sign-on functionality by integrating external authentication systems, e.g. the ELIXIR AAI, ORCID and Google.

## DATA AVAILABILTY

The provided data can be freely downloaded in various formats (e.g. CSV, JSON, PDF) without restrictions, except that the origin of the data has to be properly cited when used in other works. Registration is necessary to access data through the RESTFul API, but registration is free of charge.
